# Microbial aetiology, outcomes, and costs of hospitalisation for community-acquired pneumonia; an observational analysis

**DOI:** 10.1186/1471-2334-14-335

**Published:** 2014-06-17

**Authors:** Simone MC Spoorenberg, Willem Jan W Bos, Rik Heijligenberg, Paul GP Voorn, Jan C Grutters, Ger T Rijkers, Ewoudt MW van de Garde

**Affiliations:** 1 Department of Internal Medicine, St Antonius Hospital, P.O. Box 2500, 3430 EM, Nieuwegein, The Netherlands; 2 Department of Internal Medicine, Gelderse Vallei Hospital, P.O. Box 9025, 6710 HN, Ede, The Netherlands; 3 Department of Medical Microbiology and Immunology, St Antonius Hospital, P.O. Box 2500, 3430 EM, Nieuwegein, The Netherlands; 4 Department of Pulmonology, St Antonius Hospital, P.O. Box 2500, 3430 EM, Nieuwegein, The Netherlands; 5 Department of Pulmonology, University Medical Centre Utrecht, P.O. Box 85500, 3508 GA, Utrecht, The Netherlands; 6 Department of Sciences, Roosevelt Academy, P.O. Box 94, 4330 AB, Middelburg, The Netherlands; 7 Division of Pharmacoepidemiology and Clinical Pharmacology, University of Utrecht, P.O. Box 80125, 3508 TC, Utrecht, The Netherlands; 8 Department of Clinical Pharmacy, St Antonius Hospital, P.O. Box 2500, 3430 EM, Nieuwegein, The Netherlands

**Keywords:** Pneumonia, Bacterial infection, Health economist, Respiratory infection

## Abstract

**Background:**

The aim of this study was to investigate the clinical outcome and especially costs of hospitalisation for community-acquired pneumonia (CAP) in relation to microbial aetiology. This knowledge is indispensable to estimate cost-effectiveness of new strategies aiming to prevent and/or improve clinical outcome of CAP.

**Methods:**

We performed our observational analysis in a cohort of 505 patients hospitalised with confirmed CAP between 2004 and 2010. Hospital administrative databases were extracted for all resource utilisation on a patient level. Resource items were grouped in seven categories: general ward nursing, nursing on ICU, clinical chemistry laboratory tests, microbiology exams, radiology exams, medication drugs, and other.linear regression analyses were conducted to identify variables predicting costs of hospitalisation for CAP.

**Results:**

*Streptococcus pneumoniae* was the most identified causative pathogen (25%), followed by *Coxiella burnetii* (6%) and *Haemophilus influenzae* (5%). Overall median length of hospital stay was 8.5 days, in-hospital mortality rate was 4.8%.

Total median hospital costs per patient were €3,899 (IQR 2,911-5,684). General ward nursing costs represented the largest share (57%), followed by nursing on the intensive care unit (16%) and diagnostic microbiological tests (9%). In multivariate regression analysis, class IV-V Pneumonia Severity Index (indicative for severe disease), *Staphylococcus aureus*, or *Streptococcus pneumonia* as causative pathogen, were independent cost driving factors. *Coxiella burnetii* was a cost-limiting factor.

**Conclusions:**

Median costs of hospitalisation for CAP are almost €4,000 per patient. Nursing costs are the main cause of these costs.. Apart from prevention, low-cost interventions aimed at reducing length of hospital stay therefore will most likely be cost-effective.

## Background

Community-acquired pneumonia (CAP) is one of the most common infectious diseases worldwide; in the developed world, CAP, combined with influenza, is the primary cause of death due to infection [1]. Incidence of CAP is high in young children, then decreases and in adults, again increases with age; consequently, CAP carries a high burden, in particular in the elderly [2,3]. As 20-40% of all CAP episodes in the elderly are treated in-hospital [4], hospital admissions for pneumonia result in considerable health care costs [2,5]. In 1997, these costs were estimated to be €115 million in Spain [6] and almost £400 million in the United Kingdom [7]. With the post Second World War generation approaching senescence and the rise of life expectancy in general, the number of hospital admissions for pneumonia and associated health care costs will continue to rise [8].

Many studies have been conducted to assess the effect of interventions with the aim of reducing the risk and improving the outcome of CAP. For instance, the introduction of vaccination against Influenza has reduced severity and mortality of secondary pneumonia [9], adjunctive therapies such as corticosteroids have been shown to reduce length of hospital stay [10] and some studies showed lowering of incidence and mortality of pneumococcal pneumonia in nursing home residents through use of pneumococcal vaccines [11] although others found no protective effect [12-14]. In order to determine overall cost-effectiveness of such interventions, knowledge of the association between microbial aetiology, outcomes, and costs of CAP is indispensable.

The objectives of the present study were to analyse microbial aetiology and clinical outcomes of a large cohort of patients hospitalised for CAP, to determine individual hospital resource utilization, to quantify total costs of hospitalisation for CAP and to explore possible associations between microbial aetiology and the corrsponding costs.

## Methods

### Patients

Patients with CAP above 18 years of age admitted to the St. Antonius Hospital in Nieuwegein or the Gelderse Vallei Hospital in Ede (both teaching hospitals in the Netherlands), between October 2004 and August 2006 (n = 201), and between November 2007 and September 2010 (n = 304), who participated in two consecutive clinical studies were enrolled. The first study was a prospective cohort study on clinical characteristics and polymorphisms in innate immunity genes in patients with CAP; the second study was a placebo controlled double blind randomized clinical trial evaluating dexamethasone as adjunctive therapy (NCT00471640). In both studies, the same clinical inclusion criteria were used and patient characteristics of the patients studied resembled data from another large CAP cohort (over 20,000 patients admitted to hospital for pneumonia) from the same time period [15]. CAP was defined as a new pulmonary infiltrate on chest radiograph, in combination with at least two of the following criteria: cough, sputum production, temperature above 38°C or below 35°C, auscultatory findings consistent with pneumonia, C-reactive protein concentration of more than 15 mg/L, and white blood cell count of above 10 × 10^9^ cells/L or below 4 × 10^9^ cells/L, or >10% of rods in leukocyte differentiation. Patients who were immunocompromised, had been directly admitted to the intensive care unit (ICU), or who had received immunosuppressive therapy (including the use of >20 mg prednisone equivalent per day for >3 days) were excluded. More detailed inclusion and exclusion criteria are described elsewhere [10,16]. Comorbidities were recorded of each patient and pneumonia severity index (PSI) score was calculated on admission. The present study has been approved by the Medical Ethical Committees of the St. Antonius Hospital (Nieuwegein) and the Gelderse Vallei Hospital (Ede), both in The Netherlands.

### Microbial aetiology

At least two sets of separate blood and sputum samples of each patient were Gram stained and cultured. *Streptococcus (S.) pneumoniae* cultured from either sputum or blood was serotyped by the Quellung reaction. Moreover, sputum samples were analysed with TaqMan real-time polymerase chain reactions (PCRs) in order to detect DNA of *Mycoplasma (M.) pneumoniae*, *Legionella (L.) pneumophila*, *Coxiella (C.) burnetii*, and *Chlamydophila* species. Antigen testing of *S. pneumoniae* and *L. pneumophila* was performed in urine samples. Furthermore, pharyngeal swabs were taken for viral culture and viral PCR. Finally, patients were analysed for a serotype specific rise in *S. pneumoniae* antibodies when two blood samples (one drawn at admission and one after discharge) were available. Antibodies against pneumococcal polysaccharides were measured on a Luminex platform (Luminex Corporation, Austin, TX), using a quantitative multiplex immunoassay: the xMAP pneumococcal immunity panel. More detailed information can be found elsewhere [17].

If both a bacterium and virus were detected in a patient, the bacterial species was classified as the causative pathogen. If two different bacterial species were identified, the pathogen known to most likely cause CAP was considered causative. For the purpose of this study, aetiological agents were classified into ten groups: the first seven groups consist of the most frequently identified bacteria (*S. pneumoniae*, *C. burnetii, Haemophilus (H.) influenzae, L. pneumophila, Chlamydophila* species, *M. pneumoniae,* and *Staphylococcus aureus*), group eight contains remaining bacteria (‘Other pathogen’), group nine comprises viruses (‘Viral pathogen’), and the last group consists of CAPs with unidentified aetiology (‘No pathogen identified’).

### Clinical outcomes

ICU admission during hospitalisation, length of stay, in-hospital mortality, 30-day and one-year mortality were documented for each patient.

### Resource utilization and cost calculation

Hospital administrative databases were extracted for all resource utilisation on a patient level. Resource items were grouped in seven categories: general ward nursing, nursing on ICU, clinical chemistry laboratory tests, microbiology exams, radiology exams, medication drugs, and other. Except for nursing, only resources plausibly related to pneumonia treatment were selected. For example, medication drug use only included antibiotics, analgesics, bronchodilators, sedatives, blood products and antithrombotic drugs. The category “other” comprised physical therapy sessions, electro and echocardiograms, bronchoscopy and laryngoscopy and invasive empyema diagnostic and treatment procedures. For duration of nursing, the unit of measurement was number of days.

Total costs per patient were calculated by summing the number of resources multiplied by the costs per item. Costs per resource item were based on the National Diagnosis Treatment Combination rates valued in 2011 or 2012 [18], except for nursing costs during hospital stay and costs of medication drugs. Nursing costs for general ward and ICU stay were based on mean costs per hospital unit prices belonging to diagnostic treatment combination code 401 (‘pneumonia’) for the year 2011. Costs of drugs were based on the lowest medication drug price according to the College for Health Insurance website valued in 2012 [19]; if not available on this website, the hospital’s purchase price was recorded.

### Data analyses

Overall, descriptives were stated as number (%), mean (standard deviation (SD)) or median (interquartile range (IQR)), and compared using independent samples T-test, Chi-square test, or Mann–Whitney U test, where appropriate. Kruskall Wallis test was used to assess overall differences in length of stay and costs between aetiologic groups.

To identify variables predicting costs of hospitalisation for CAP, linear regression analyses were conducted with log-transformed data. Costs were log-transformed to correct for skewness of the data. First, the following variables were examined in a univariate model (with reference group): male gender (female), chronic obstructive pulmonary disease (no chronic obstructive pulmonary disease), congestive heart failure (no congestive heart failure), diabetes mellitus (no diabetes mellitus), PSI classes IV-V *(*classes I-III), and admission in ‘Gelderse Vallei Hospital’ (St. Antonius Hospital). The ten aetiologic groups were included separately in the model; reference value per group was composed of the other nine aetiologic groups. Subsequently, variables significant in univariate models (p < 0.10) were inserted in a multivariate model, applying a backwards elimination technique retaining variables with a p-value < 0.10. For the final model, effects (costs) were stated as beta with corresponding standard error for each independent variable. Data were analysed with SPSS statistical software for Windows, version 21.0. For all analyses, a p-value of <0.05 was considered statistically significant.

## Results

A total of 505 patients with CAP were subject in this study, with a mean age of 63.4 ± 18.0 years and a male/female ratio of 1.4/1. Patient characteristics are presented in Table 1.

**Table 1:** Characteristics of 505 patients hospitalised with community-acquired pneumonia

**Characteristics**	**All patients (*n*** **= 505)**
Age in years (SD)	63.4 (18.0)
Male sex (%)	295 (58.4)
Comorbidities (%)	
Chronic obstructive pulmonary disease	98 (19.4)
Congestive heart failure	68 (13.5)
Renal disease	40 (7.9)
Diabetes mellitus	77 (15.2)
Liver disease	2 (0.4)
Pneumonia Severity Index class I-III (%)	279 (55.2)
Pneumonia Severity Index class IV-V (%)	226 (44.8)
Pathogens (%)	
*Streptococcus pneumoniae*	124 (24.6)
*Coxiella burnetii*	28 (5.5)
*Haemophilus influenzae*	27 (5.3)
*Legionella pneumophila*	20 (4.0)
*Chlamydophila* species	16 (3.2)
*Mycoplasma pneumoniae*	9 (1.8)
*Staphylococcus aureus*	9 (1.8)
Only viral pathogen	35 (6.9)
Other pathogen	27 (5.3)
No pathogen identified	210 (41.6)
Empirical antibiotic treatment (%)	
Beta-lactam, penicillins (monotherapy)	254 (50.3)
Other beta-lactam (monotherapy)	86 (17.0)
Beta-lactam, penicillins + quinolone	34 (6.7)
Beta-lactam, penicillins + macrolides	33 (6.5)
Other beta-lactam + aminoglycoside	20 (4.0)
Other beta-lactam + quinolone	16 (3.2)
Quinolone (monotherapy)	11 (2.2)
Macrolides, lincosamides and streptogramins (monotherapy)	11 (2.2)
Beta-lactam, penicillin + aminoglycoside	7 (1.4)
Other beta-lactam, penicillin + macrolides	7 (1.4)
Sulfanomides and trimethoprim (monotherapy)	6 (1.2)
Tetracyclines (monotherapy)	6 (1.2)
Other	14 (2.8)
Outcomes	
Length of hospital stay (IQR)	8.5 (6.0-13.0)
Intensive care unit admission (%)	38 (7.5)
In-hospital mortality (%)	24 (4.8)
30-Day mortality (%)	26 (5.1)
One-year mortality (%)	73 (14.5) ^⟂^

Data are presented as number (%), mean (SD) or median (IQR).

*Abbreviations*: *IQR* interquartile range, *SD* standard deviation.

^⟂^7 patients were lost to follow-up.

### Aetiology and clinical outcomes

In 294/505 (58.2%) patients, a causative pathogen was identified. Table 2 lists the microbiological test results of most frequently identified pathogens. Overall, *S. pneumoniae* was most prevalent (124/505, 24.6%). In 51 of these 124 patients, *S. pneumoniae* serotyping could be performed. Type 1 was the most common serotype. A complete overview of the pneumococcal serotypes is given in Additional file 1: Table S1. In 43/505 patients a mixed infection was found. No penicillin resistant *S. pneumoniae*, methycillin-resistant *Staphylococcus aureus* or multi-resistant gram-negative pathogens were identified.

**Table 2:** Microbiology tests results of 505 patients hospitalised with community-acquired pneumonia

	**Sputum culture**	**Sputum PCR**	**Blood culture**	**Blood PCR**	**Urinary antigen test**	**Serology**
*S. pneumoniae* n *=* 124	46	-	42	-	78	1
*Haemophilus influenzae* n = 27	25	1	-	-	-	1
*Legionella pneumophila* n = 20	1	3	-	-	15	6
*Mycoplasma pneumoniae* n = 9	-	4	-	-	-	7
*Coxiella burnetii* n = 28	1	12	2	12	-	20
*Chlamydophila* spp. n = 16	-	8	-	-	-	12
*Staphylococcus aureus* n = 9	8	-	1	-	-	-
Other pathogen n = 27	23	-	5	-	-	-
Viral pathogen n = 35	3	2	-	-	-	19

*Abbreviations*: *n* number, *S. pneumoniae Streptococcus pneumoniae*, *spp* species.

Clinical outcomes categorized by aetiology group are listed in Table 3. Overall, LOS differed significantly between the major aetiological groups (p < 0.001). CAP caused by either *M. pneumoniae* or *C. burnetii* was associated with a significantly shorter LOS compared to the other aetiologic groups (p:0.007 and p < 0.001, respectively), while *S. pneumoniae* CAPs resulted in a significantly longer duration of hospital stay (p:0.03).

**Table 3:** Clinical outcomes per pathogen of 505 patients hospitalised with community-acquired pneumonia

	**Length of hospital stay (IQR)**	**ICU admission (%)**	**In-hospital mortality (%)**	**30-day mortality (%)**	**One-year mortality (%)**
*Streptococcus pneumoniae* (n = 124)	8.5 (6.5-14.9)	10 (8.1)	4 (3.2)	4 (3.2)	12 (9.7)
*Coxiella burnetii* (n = 28)	5.5 (3.5-7.5)	0	0	1 (3.6)	1 (3.6)
*Haemophilus Influenzae* (n = 27)	9.0 (7.5-14.0)	2 (7.4)	0	0	3 (11.1)
*Legionella pneumophila* (n = 20)	11.0 (6.5-17.0)	3 (15.0)	1 (5.0)	1 (5.0)	2 (10.0)
*Chlamydophila* species (n = 16)	8.5 (6.6-13.3)	2 (12.5)	0	0	1 (6.3)
*Mycoplasma pneumoniae* (n = 9)	5.0 (4.5-7.3)	0	0	0	0
*Staphylococcus aureus (*n = 9)	10.5 (7.3-14.5)	1 (11.1)	3 (33.3)	3 (33.3)	4 (44.4)
Other pathogen (n = 27)	8.0 (5.0-15.8)	7 (25.9)	2 (7.4)	2 (7.4)	9 (33.3)
Viral pathogen (n = 35)	8.5 (6.3-13.5)	1 (2.9)	2 (5.7)	3 (8.6)	6 (17.1)
No pathogen found (n = 210)	8.5 (5.5-12.6)	12 (5.7)	12 (5.7)	12 (5.7)	35 (16.7)

Data are presented as number (%) or median (IQR). For calculation of median length of stay patients who died during admission where excluded from the analysis.

A*bbreviations*: *ICU* intensive care unit, *IQR* interquartile range*, n* number.

### Hospital costs

For 361/505 (71.5%) of the patients complete resource utilization data were available for analysis. The clinical characteristics of the 144 patients who could not be included, as compared to the included patients can be found in Additional file 1: Table S2.

### Total costs

**Figure 1:**
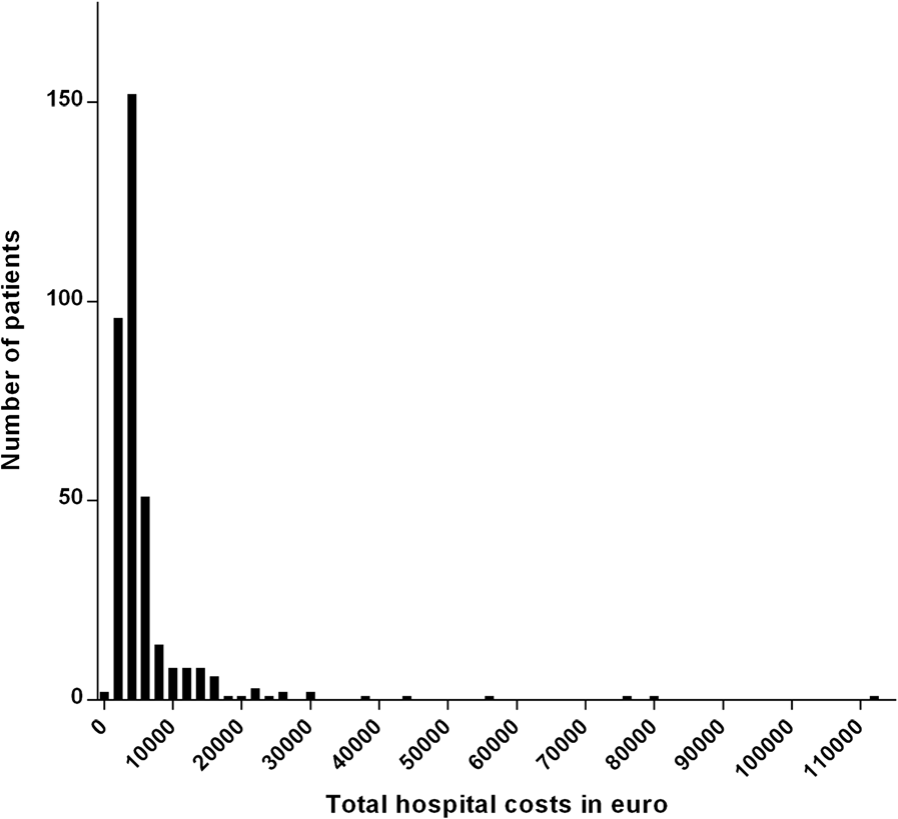
Distribution of total hospital costs in 361 patients hospitalised with community-acquired pneumonia.

**Figure 2:**
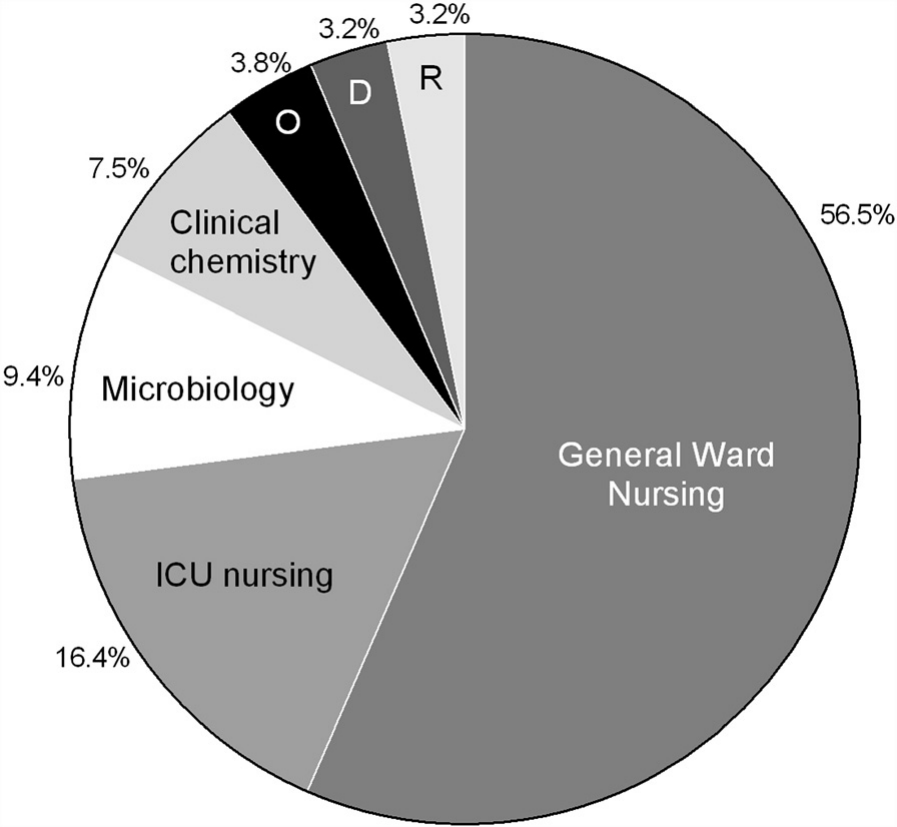
**Distribution of costs of 361 patients hospitalised with community-acquired pneumonia expressed by their seven major resource categories.** Legend: Abbreviations: D, drugs; ICU, intensive care unit nursing; O, other; R, radiology exams.

Table 4 lists the top 10 most frequent and the top 10 most expensive resource items. In the Additional file 1, the top 5 most frequent used items for each individual category can be found in Table S3.Figure 1 shows the total distribution of hospital costs per patient. Total median hospital costs per patient were €3,899 (IQR 2,911-5,684) with minimum costs of €901 and maximum costs of €112,634. Figure 2 shows the share per category in the total costs: general ward nursing represented the largest share (56.5%), followed by nursing on ICU (16.4%) and diagnostic microbiology exams (9.4%).

**Table 4:** Top 10 most frequent and top 10 most expensive resource items with prices in euro

	**Resource**	**Mean frequency per patient**	**Price per item (in euro)**
	**10 Most frequent resource items**		
**1**	Tissue obtainment (microbiology and clinical chemistry)	18.1	13.73
**2**	Antibodies against any pathogen by using complement fixation test of haemagglutination inhibition essay	10.4	4.84
**3**	General ward nursing (one day)	9.4	375.00
**4**	Sodium	8.2	1.76
**5**	Potassium	8.2	1.76
**6**	Creatinine	6.6	1.76
**7**	Glucose	6.4	1.76
**8**	Leukocytes	6.1	1.76
**9**	C-reactive protein	6.0	4.84
**10**	Urea	5.9	1.76
	**10 Most expensive resource items**		
**1**	Intensive care unit nursing (one day)	0.6	1,730.00
**2**	Surgical treatment of thorax empyema	0.2	1,084.75
**3**	Laryngoscopy	0.0	999.00
**4**	Microbiological determination on isolated DNA/RNA	0.0	867.61
**5**	General ward nursing (one day)	9.4	375.00
**6**	Flexible bronchoscopy	0.2	209.25
**7**	DNA/RNA amplification (qualitative)	<0.1	208.60
**8**	Computer tomography of thorax	0.2	195.83
**9**	Immunopathologic research	<0.1	109.57
**10**	Computer tomography airways	<0.1	160.01

### Costs categorized per aetiological group

Overall, total hospital costs differed between the 10 aetiological groups (p:0.002); costs for hospitalisation of CAP caused by *C. burnetii* were significantly lower (p < 0.001), while hospitalisation of patients with *S. pneumoniae* as causative agent represents significantly higher costs (p:0.03) compared to other aetiologies. For *M. pneumoniae* and *Staphylococcus aureus* a trend towards respectively lower and higher costs (p: 0.10 and p:0.08, respectively) was observed.

**Figure 3:**
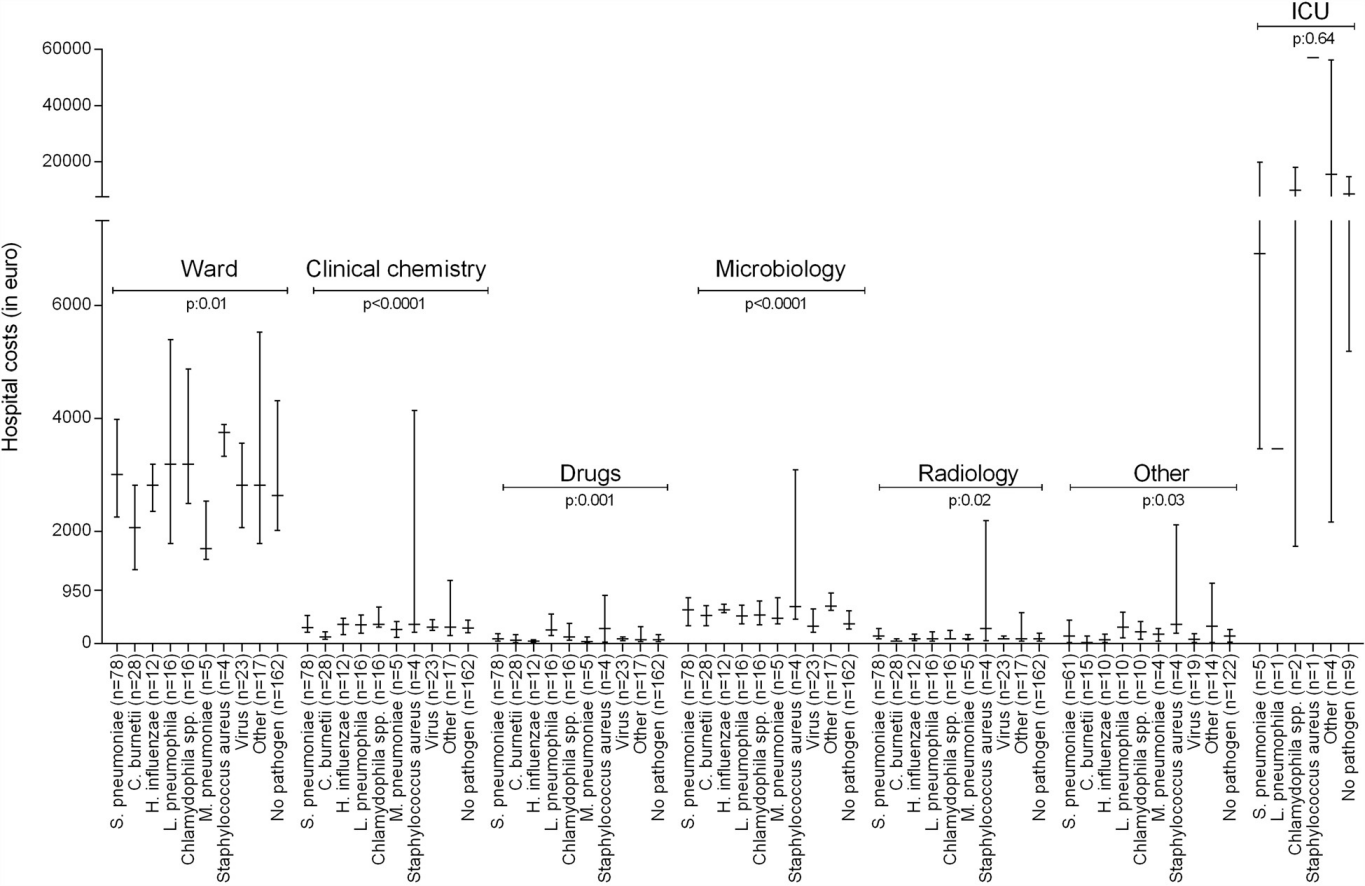
**Median hospital costs in euro with interquartile range per aetiology differentiated by resource group.** Legend: Abbreviations: *C. burnetii, Coxiella burnetii; H. influenzae, Haemophilus influenzae;* ICU, intensive care unit; *L. pneumophila, Legionella pneumophila; M. pneumoniae, Mycoplasma pneumoniae;* n, number; spp, species; *S. pneumoniae*, *Streptococcus pneumoniae*.

Figure 3 shows median costs of each aetiologic group subdivided into the seven resource categories. Raw numbers of this figure can be found in Additional file 1: Table S4. Overall, costs for general ward nursing, microbiology exams, clinical chemistry laboratory tests, medication drugs, and radiologic exams all differed between the aetiological groups. On an individual pathogen level, CAP caused by *C. burnetii* was lower in costs for nursing, clinical chemistry tests, and radiological examinations compared to most of the other aetiological groups. Costs of medication were specifically high in patients with *L. pneumophila* pneumonia. CAP caused by *Staphylococcus* a*ureus* was higher in costs for nursing and CAP caused by *S. pneumoniae* was more expensive in radiological examinations. Additional file 1: Table S5A to S5G shows details of this aetiological subgroup analysis.

### Costs per *S. pneumoniae* serotype

As *S. pneumoniae* is the most frequent identified pathogen in CAP, costs of serotypes were explored grouped per pneumococcal vaccine available in the European Union (results presented in Additional file 1: Table S6). Total costs of hospitalisation were not higher for patients with CAP caused by the serotypes present in the different vaccines compared to patients infected by pneumococcal serotypes not included in these vaccines.

### Identification of cost driving factors

To identify cost driving factors, a multivariable linear regression model was constructed. Table 5 lists the variables included in the final model together with their corresponding regression coefficients. *Staphylococcus aureus*, high PSI score (classes IV and V), and *Streptococcus pneumoniae* were all independent cost driving factors, increasing total costs of hospitalisation by 98%, 43%, and 18% respectively. *Coxiella burnetii* decreased total costs of hospitalisation by 35%.

**Table 5:** Multivariable linear regression model to predict total costs of hospitalisation in 361 patients with community-acquired pneumonia

	**Crude analysis**			**Multivariable analysis**		
**Independent variables**	**B**	**SE**	**p-value**	**B**	**SE**	**p-value**
CONSTANT				**3.558**	**0.023**	**<0.001**
Male gender	0.009	0.032	0.77			
Chronic obstructive pulmonary disease	0.009	0.044	0.83			
Congestive heart failure	**0.083**	**0.045**	**0.06**			
Chronic renal disease	0.077	0.057	0.17			
Diabetes mellitus	0.047	0.045	0.30			
Pneumonia Severity Index classes IV-V	**0.176**	**0.030**	**<0.001**	**0.158**	**0.030**	**<0.001**
Hospital ‘Gelderse Vallei’	−0.063	0.042	0.13			
Pathogens:						
*Streptococcus pneumoniae*	**0.070**	**0.038**	**0.07**	**0.067**	**0.036**	**0.07**
*Coxiella burnetii*	**−0.198**	**0.058**	**0.001**	**−0.129**	**0.056**	**0.02**
*Haemophilus influenzae*	−0.047	0.087	0.59			
*Legionella pneumophila*	0.060	0.076	0.43			
*Chlamydophila* species	0.087	0.076	0.25			
*Mycoplasma pneumoniae*	−0.179	0.134	0.18			
*Staphylococcus aureus*	**0.358**	**0.148**	**0.02**	**0.362**	**0.140**	**0.01**
Viral pathogen	−0.046	0.064	0.48			
Other pathogen	**0.163**	**0.073**	**0.03**	**0.131**	**0.070**	**0.06**
No pathogen found	−0.034	0.031	0.28			

Prices are log-transformed and stated in euro.

*Abbreviations*: *B* beta coefficient, *SE* standard error.

Bold numbers are included in the final model (p < 0.10).

## Discussion

In the past years, many studies have been conducted aiming at finding new strategies to lower incidence and improve clinical outcomes of CAP. To determine cost-effectiveness of these strategies, knowledge about causing microorganisms, clinical outcomes, and related costs is needed. To our knowledge, this is the first study that studies the potential associations between costs of hospitalisation for CAP and its microbial aetiology. The main finding in the present study is that costs related to hospitalisation for CAP show great variation between patients, and CAP caused by *S. pneumoniae* and *Staphylococcus aureus* is associated with significantly higher costs, mainly due to longer duration of hospital stay.

In this study, *S. pneumoniae* was confirmed as the most prevalent causative pathogen in CAP (24.6%). Compared to other aetiological groups, median LOS (8.5 days), rate of ICU admission (8%), and one-year mortality (9.7%) were relatively higher for pneumonia caused by *S. pneumoniae*, despite the relative younger age of patients of this aetiological group (60.4 ± 19.0 years versus 64.4 ± 17.6 years, p:0.033). These findings are in accordance with other CAP studies that also reported higher disease severity and increased need for ICU admission in *S. pneumoniae* pneumonia [20,21]. In agreement with these findings, we showed *S. pneumoniae* to be an independent cost-driving factor (on average plus 18% per hospitalisation).

Interestingly, *Staphylococcus aureus* could also be identified as an independent cost driving factor. CAPs caused by this pathogen were associated with a longer LOS and a higher mortality rate as well. This unfavourable outcome might be explained by the difficulty of treating *Staphylococcus aureus* pulmonary and systemic infections*.* Recently, Restrepo et al. have reported that late ICU admission versus early ICU admission is more prevalent in cases of CAP caused by *Staphylococcus aureus*, which aligns with the higher mortality rate observed in our study [22].

In our study, median total costs of hospitalisation were almost €4,000 per patient. These expenditures are higher compared to similar studies performed in Germany and Spain (median costs of €1,362 [23], €1,683 [24] and €1,553 [25], respectively), but lower than reported in a study from the United Kingdom (£1,700-5,100, depending on length of stay [7]) and a European study (US$6,530 in a secondary-level hospital in the Netherlands and US$8,444 in a teaching hospital) [26]. The most likely explanation for these discrepancies in hospital costs are expected to be differences in registration, and individual resource item prices. Furthermore, diagnostic and treatment standards might differ between countries, leading to other price calculations. The recent study of Ostermann et al., however, showed no large differences in mean total duration of hospital stay for CAP between several EU countries (range 9.6-15.0 days) [26]. Unfortunately, most published studies do not indicate prices of individual resource items, which makes detailed comparisons between studies very difficult. Besides this, none of the available studies in literature included aetiological groups in their analyses, further limiting the possibility of a relative comparison with our study findings at this moment.

A further relevant finding in our study was that 57% of the total costs of hospitalisation is due to general ward nursing. This finding is in accordance with other costs studies [27,28]. The latter is also reflected by *C. burnetii*, causing a relatively milder course of the disease and a significant shorter duration of hospital stay, being identified as an independent cost limiting factor in the multivariable model.

In the present study, costs of medication represented a very small part of the total costs of hospitalisation (on average 3.2%). This means that policies aiming at an early intravenous to oral switch of antimicrobial treatment will not result in substantial cost-savings by reducing drug-expenses; costs might be reduced if the switch resulted in earlier hospital discharge. Medication costs for pneumonia caused by *Legionella pneumophila* appeared significantly higher compared to other aetiological groups. This is most likely caused by a higher ICU admission rate for these pneumonias and linked to the use of specific drugs such as fresh frozen plasma and sedatives.

This study has several strengths. First, we were able to identify the causative pathogen in a large number of patients enabling comparisons between aetiological groups. Second, we analysed resource utilization on an individual patient level. Third, data of two hospitals were studied (showing no differences) adding to the external validity of the findings. Besides this, the characteristics of the patients studied resemble data from another large nationwide CAP cohort from the Netherlands further adding to the generalisability of the findings [15].

There are also limitations that need to be addressed. First, due to missing data in some resources categories, not all 505 patients could be included in the overall cost analyses. This was due to being unable to retrieve some resource use from the years 2004 until 2006. We consider, however, that this has no impact on the validity of the findings because the more recent years are fully included , making the total costs of hospitalisation representative for the present standard of care for CAP. A further reassuring factor is that the comparison of patient characteristics and clinical outcomes of the 361 patients included in the analyses with the 144 patients not included, showed no large differences (see Additional file 1: Table S2). However, the lower number of patients available for analysis resulted in some aetiological subgroups becoming rather small.

Another limitation is that patients directly admitted to the ICU were absent in the study cohorts used. In the most recent cohort, 25 of the 817 eligible patients (3%) were not included due to direct ICU admission. This phenomenon could have lead to an underestimation of the absolute costs of hospitalisation for CAP. However, given this low percentage, we expect this effect to be rather small. Furthermore, it is very unlikely to have biased the relative costs per pathogen.

Finally, we cannot rule out that the costs related to microbiology exams are overestimated (9% share of total costs of hospitalisation). We studied patients who had participated in clinical studies in which a large panel of microbiological tests had been performed to maximize pathogen identification. However, presuming this resulted in a 50% increase in microbiology costs, decreasing these costs by 50% influences the total costs by less than 5%. In the present study, 58.2% of the causative pathogens could be identified, which is relatively high as compared to other studies [9].

## Conclusions

In conclusion, in the present study we have shown that the total costs of hospitalisation for CAP vary considerably between patients and this variation can be largely explained by differences in length of hospital stay. Increased disease severity, and *S. pneumoniae* and *Staphylococcus aureus* as causative pathogens, are independent cost driving factors. This suggests, from a cost perspective, to focus further research on better in-hospital treatment and prevention of CAP caused by these pathogens. As standards of care and individual resource item prices are expected to differ between countries, further study in other countries should be performed to confirm the results of this study.

## Consent

From all patients written informed consent was obtained in both studies.

## Abbreviations

C. burnetii: Coxiella burnetii; CAP: Community-acquired pneumonia; H. influenzae: Haemophilus influenzae; ICU: Intensive care unit; IQR: Interquartile range; L. pneumophila: Legionella pneumophila; M. pneumoniae: Mycoplasma pneumoniae; PCR: Polymerase chain reaction; PSI: Pneumonia severity index; S. pneumoniae: Streptococcus pneumoniae; SD: Standard deviation.

## Competing interests

The department of Pharmacoepidemiology and Clinical Pharmacology, Utrecht Institute for Pharmaceutical Sciences, has received unrestricted research funding from the Netherlands Organisation for Health Research and Development (ZonMW), the Dutch Health Care Insurance Board (CVZ), the Royal Dutch Pharmacists Association (KNMP), the private-public funded Top Institute Pharma (http://www.tipharma.nl, includes co-funding from universities, government, and industry), the EU Innovative Medicines Initiative (IMI), EU 7th Framework Program (FP7), the Dutch Medicines Evaluation Board, the Dutch Ministry of Health and industry (including GlaxoSmithKline, Pfizer, and others).

## Authors’ contributions

SMCS performed the data analysis and interpretation and drafted the manuscript. WJWB and GTR were involved in data interpretation and revised the manuscript critically for important intellectual content. RH, GPV and JCG revised the manuscript critically. EMWG designed the study, was involved in data analysis and interpretation and revised the manuscript critically for important intellectual content. All authors had full access to the data and can take responsibility for the integrity of the data and the accuracy of the data analysis. All authors approved the final version of the manuscript. EMWG is the guarantor. All authors read and approved the final manuscript.

## Pre-publication history

The pre-publication history for this paper can be accessed here:

http://www.biomedcentral.com/1471-2334/14/335/prepub

## Supplementary Material

Additional file 1 **Web appendix: Table S1.** Serotype distribution in 51 patients with *Streptococcus pneumoniae* pneumonia. **Table S2**. Patient characteristics of 361 patients included in the pneumonia cost analyses compared to patients not included in the cost analyses. **Table S3**. Top 5 most frequently used items for each resource group. **Table S4**. Median hospital costs in euro with interquartile range per aetiology differentiated by resource group. **Table S5**. P-values of the seven resource categories subdivided by ten aetiologic groups. **Table S6**. Median total costs of hospitalisation in euro of pneumococcal vaccines serotypes compared with costs of non-vaccine serotypes, with serotyping determined in two manners. Click here for file
