# Supplementary material for: Microbial aetiology, outcomes, and costs of hospitalisation for community-acquired pneumonia; an observational analysis
Source: BMC Infect Dis. 2014 Jun 17;14:335. doi: 10.1186/1471-2334-14-335 (PMC4078020; doi:10.1186/1471-2334-14-335)
Supplement: Additional file 1 — Web appendix: Table S1. Serotype distribution in 51 patients with Streptococcus pneumoniae pneumonia. Table S2. Patient characteristics of 361 patients included in the pneumonia cost analyses compared to patients not included in the cost analyses. Table S3. Top 5 most frequently used items for each resource group. Table S4. Median hospital costs in euro with interquartile range per aetiology differentiated by resource group. Table S5. P-values of the seven resource categories subdivided by ten aetiologic groups. Table S6. Median total costs of hospitalisation in euro of pneumococcal vaccines serotypes compared with costs of non-vaccine serotypes, with serotyping determined in two manners. [file 1471-2334-14-335-S1.doc]

**Additional file 1**

**WEB APPENDIX**

**TABLE S1.** Serotype distribution in 51 patients with *Streptococcus pneumoniae* pneumonia.

| *Streptococcus pneumoniae*  Serotype | Serotype incorporated in 7, 10, 13  or 23-valent vaccine? | Number of  patients (%) |
| --- | --- | --- |
| type 1 | 10, 13, 23 | 9 (17.6) |
| type 14 | 7, 10, 13, 23 | 5 (9.8) |
| type 8 | 23 | 5 (9.8) |
| type 9* |  | 5 (9.8) |
| type 3 | 13, 23 | 4 (7.8) |
| type 4 | 7, 10, 13, 23 | 3 (5.9) |
| type 7F | 10, 13, 23 | 3 (5.9) |
| type 9V | 7, 10, 13, 23 | 3 (5.9) |
| type 11A | 23 | 2 (3.9) |
| type 16F | None | 2 (3.9) |
| type 19A | 13, 23 | 2 (3.9) |
| type 7* |  | 2 (3.9) |
| type 10A | 23 | 1 (2.0) |
| type 19F | 7, 10, 13, 23 | 1 (2.0) |
| type 22* |  | 1 (2.0) |
| type 22F | 23 | 1 (2.0) |
| type 23B | None | 1 (2.0) |
| type 6B | 7, 10, 13, 23 | 1 (2.0) |
| Total |  | 51 (100%) |

Abbreviation: 7, 7-valent conjugate vaccine; 10, 10-valent conjugate vaccine; 13,13-valent conjugate vaccine; 23, 23-valent polysaccharide vaccine.

7-valent conjugate vaccine contains serotypes 4, 6B, 9V, 14, 18C, 19F, and 23F;

10-valent conjugate vaccine contains serotypes 1, 4, 5, 6B, 7F 9V, 14, 18C, 19F, and 23F;

13-valent conjugate vaccine contains serotypes 1, 3, 4, 5, 6A, 6B, 7F, 9V, 14, 18C, 19A, 19F, and 23F;

23-valent polysaccharide vaccine contains serotypes 1, 2, 3, 4, 5, 6B, 7F, 8, 9N, 9V, 10A, 11A, 12F, 14, 15B, 17F, 18C, 19F, 19A, 20, 22F, 23F, and 33F.

* Indicates subtype is not defined.

**TABLE S2. Patient characteristics of 361 patients included in the pneumonia cost analyses compared to patients not included in the cost analyses.**

| Characteristics | Patients  in cost analysis  (*n* = 361) | Patients not  in cost analysis  (*n* = 144) | *p-*value |
| --- | --- | --- | --- |
| Age in years (IQR) | 66 (55-79) | 68 (52-76) | 0.75 |
| Male sex (%) | 207 (57.3) | 88 (61.1) | 0.44 |
| Comorbidities (%)  COPD  Congestive heart failure  Chronic renal disease  Diabetes mellitus  Liver disease | 55 (15.2)  51 (14.1)  30 (8.3)  52 (14.4)  2 (0.6) | 43 (29.9)  17 (11.8)  10 (6.9)  25 (17.4)  0 | <0.001*  0.49  0.61  0.40  1.00 |
| PSI classes I-III (%)  PSI classes IV-V (%) | 195 (54.0)  166 (46.0) | 84 (58.3)  60 (41.7) | 0.38 |
| Pathogens (%)  Streptococcus pneumoniae  Coxiella burnetii  Haemophilus influenzae  Legionella pneumophila  Chlamydophila species  Mycoplasma pneumoniae  *Staphylococcus aureus*  Only viral pathogen  Other pathogen  No pathogen found | 78 (21.6)  28 (7.8)  12 (3.3)  16 (4.4)  16 (4.4)  5 (1.4)  4 (1.1)  23 (6.4)  17 (4.7)  162 (44.9) | 46 (31.9)  0  15 (10.4)  4 (2.8)  0  4 (2.8)  5 (3.5)  12 (8.3)  10 (6.9)  48 (33.3) | <0.001* |
| Empirical antibiotic treatment (%) |  |  | <0.001* |
| [Beta-lactam, penicillins](http://www.whocc.no/atc_ddd_index/?code=J01C&showdescription=no) (mono) | 163 (45.2) | 91 (63.2) |  |
| Other beta-lactam (mono) | 70 (19.4) | 16 (11.1) |  |
| [Beta-lactam, penicillins](http://www.whocc.no/atc_ddd_index/?code=J01C&showdescription=no) + quinolone | 27 (7.5) | 7 (4.9) |  |
| [Beta-lactam, penicillins](http://www.whocc.no/atc_ddd_index/?code=J01C&showdescription=no) + macrolides | 26 (7.2) | 7 (4.9) |  |
| Other beta-lactam + aminoglycoside | 18 (5.0) | 2 (1.4) |  |
| Other beta-lactam + quinolone | 16 (4.4) | 0 |  |
| [Macrolides](http://www.whocc.no/atc_ddd_index/?code=J01F&showdescription=no) (mono) | 7 (1.9) | 4 (2.8) |  |
| Other beta-lactam + macrolides | 7 (1.9) | 0 |  |
| Quinolone (mono) | 6 (1.7) | 5 (3.5) |  |
| Tetracyclines (mono) | 6 (1.7) | 0 |  |
| Beta-lactam, penicillin + aminoglycoside | 5 (1.4) | 2 (1.4) |  |
| [Sulfanomides or trimethoprim](http://www.whocc.no/atc_ddd_index/?code=J01E&showdescription=no)  (mono) | 4 (1.1) | 2 (1.4) |  |
| Other | 6 (1.8) | 8 (5.6) |  |
| Outcomes  Length of hospital stay (IQR) | 7.5 (5.5-11.5) | 11 (8.0-16.0) | <0.001* |
| ICU admission (%)  In-hospital mortality (%) | 22 (6.1)  19 (5.3) | 16 (11.1)  5 (3.5) | 0.05  0.39 |
| 30-Day mortality (%) | 21 (5.8) | 5 (3.5) | 0.28 |
| One-year mortality (%) | 48 (13.3) ^⊥^ | 25 (17.4) ^‡^ | 0.26 |

Data are presented as number (%), mean (SD) or median (IQR).

Abbreviations: COPD, chronic obstructive pulmonary disease; ICU, intensive care unit; IQR interquartile range; PSI, pneumonia severity index; SD, standard deviation.

^⊥^ Missing data of 6 patients; ^‡^ Missing data of 1 patients; * Indicates a significant association (*p*-value <0.05).

**TABLE S3. Top 5 most frequently used items for each resource group.**

|  | RESOURCE | MEAN FREQUENCY PER PATIENT |
| --- | --- | --- |
|  | **GENERAL WARD NURSING** |  |
| **1** | General ward nursing day (one day) | 9.4 |
|  | **INTENSIVE CARE UNIT NURSING** |  |
| **1** | Intensive care unit nursing day (one day) | 0.6 |
|  | **CLINICAL CHEMISTRY LABORATORY TESTS** |  |
| **1** | Tissue obtainment (microbiology and clinical chemistry) | 18.1 |
| **2** | Sodium | 8.2 |
| **3** | Potassium | 8.2 |
| **4** | Creatinine | 6.6 |
| **5** | Glucose | 6.4 |
|  | **MICROBIOLOGY EXAMS** |  |
| **1** | Antibodies against any pathogen by using complement fixation test or haemagglutination inhibition assay | 10.4 |
| **2** | Susceptibility testing by using MIC determination / E-test | 4.0 |
| **3** | Aerobic and anaerobic blood culture | 2.6 |
| **4** | Antigen or toxine detection in clinical material by immunoassay | 2.6 |
| **5** | Microscopy of stained and unstained preparations | 2.4 |
|  | **RADIOLOGY EXAMS** |  |
| **1** | Chest X-ray | 3.3 |
| **2** | Computer tomography of thorax, heart and large vessels | 0.2 |
| **3** | Abdominal X-ray | 0.1 |
| **4** | Computer tomography of abdomen | 0.1 |
| **5** | Computer tomography of thorax | <0.1 |
|  | **MEDICATION DRUGS** |  |
| **1** | Paracetamol tablets | 4.8 days |
| **2** | Nadroparin subcutanous injection | 4.5 days |
| **3** | Amoxicillin-clavulanic acid for infusion | 3.0 days |
| **4** | Ipramole inhalation | 2.3 days |
| **5** | Acetylcystein inhalation | 2.0 days |
|  | **OTHER** |  |
| **1** | Fysiotherapy | 3.4 |
| **2** | Electrocardiogram | 0.8 |
| **3** | Bronchoscopy | 0.2 |
| **4** | Surgical treatment of thorax empyema | 0.2 |
| **5** | Diagnostic pleurapunction with ultrasound | <0.1 |

**TABLE S4**. Median hospital costs in euro with interquartile range per aetiology differentiated by resource group.

|  | **GENERAL WARD NURSING** | | | **CLINICAL CHEMISTRY LABORATORY TESTS** | | | **MEDICATION DRUGS** | | | **MICROBIOLOGY EXAMS** | | |
| --- | --- | --- | --- | --- | --- | --- | --- | --- | --- | --- | --- | --- |
|  | Med. | IQR | *N* | Med. | IQR | *N* | Med. | IQR | *N* | Med. | IQR | *N* |
| *Streptococcus pneumoniae* | 3,000 | (2,250-3,984) | *78* | 290 | (206-504) | *78* | 89 | (49-175) | *78* | 599 | (318-820) | *78* |
| *Coxiella burnetii* | 2,063 | (1,313-2,813) | *28* | 120 | (80-208) | *28* | 60 | (18-158) | *28* | 504 | (317-679) | *28* |
| *Hemophilus influenzae* | 2,813 | (2,344-3,188) | *12* | 342 | (165-449) | *12* | 48 | (31-70) | *12* | 602 | (553-699) | *12* |
| *Legionella pneumophila* | 3,188 | (1,781-5,391) | *16* | 334 | (188-508) | *16* | 249 | (149-527) | *16* | 493 | (352-681) | *16* |
| *Chlamydophila species* | 3,188 | (2,484-4,875) | *16* | 343 | (294-652) | *16* | 118 | (63-362) | *16* | 508 | (340-762) | *16* |
| *Mycoplasma pneumoniae* | 1,688 | (1,500-2,531) | *5* | 251 | (115-397) | *5* | 37 | (17-117) | *5* | 456 | (356-816) | *5* |
| *Staphylococcus aureus* | 3,750 | (3,328-3,891) | *4* | 346 | (200-4,144) | *4* | 271 | (30-861) | *4* | 662 | (433-3,086) | *4* |
| *Viral* | 2,813 | (2,063-3,563) | *23* | 297 | (240-428) | *23* | 90 | (53-119) | *23* | 313 | (202-616) | *23* |
| *Other* | 2,813 | (1,781-5,531) | *17* | 291 | (146-1,129) | *17* | 70 | (41-300) | *17* | 671 | (593-905) | *17* |
| *No pathogen* | 2,625 | (2,106-4,313) | *162* | 276 | (195-421) | *162* | 73 | (39-164) | *162* | 350 | (258-589) | *162* |
| **ALL** | **2,813** | **(2,063-3,938)** | **361** | **283** | **(190-425)** | **361** | **78** | **(40-185)** | **361** | **481** | **(282-693)** | **361** |

|  | **RADIOLOGY EXAMS** | | | **OTHER** | | | **ICU NURSING** | |  |
| --- | --- | --- | --- | --- | --- | --- | --- | --- | --- |
|  | Med. | IQR | *N* | Med. | IQR | *N* | Med. | IQR | *N* |
| *Streptococcus pneumoniae* | 136 | (91-272) | *78* | 140 | (18-421) | *61* | 6,920 | (3,460-19,895) | *5* |
| *Coxiella burnetii* | 45 | (45-91) | *28* | 18 | (18-137) | *15* | N.A. | N.A. | *0* |
| *Hemophilus influenzae* | 91 | (57-170) | *12* | 67 | (18-166) | *10* | N.A. | N.A. | *0* |
| *Legionella pneumophila* | 91 | (45-216) | *16* | 298 | (107-558) | *10* | 3,460 | N.A. | *1* |
| *Chlamydophila species* | 91 | (91-238) | *16* | 211 | (78-396) | *10* | 18,165 | (1,730) | *2* |
| *Mycoplasma pneumoniae* | 91 | (68-166) | *5* | 173 | (48-269) | *4* | N.A. | N.A. | *0* |
| *Staphylococcus aureus* | 272 | (56-2,186) | *4* | 345 | (187-2,118) | *4* | 57,090 | N.A. | *1* |
| *Viral* | 91 | (91-136) | *23* | 76 | (18-177) | *19* | N.A. | N.A. | *0* |
| *Other* | 91 | (45-553) | *17* | 309 | (18-1,072) | *14* | 15,570 | (2,163-56,225) | *4* |
| *No pathogen* | 91 | (45-187) | *162* | 137 | (27-252) | *122* | 8,650 | (5,190-14,705) | *9* |
| **ALL** | **91** | **(45-193)** | **361** | **137** | **(18-314)** | **269** | **7,785** | **(3,460-29,410)** | **22** |

Abbreviations: ICU, intensive care unit; IQR, interquartile range; med, median; n, number of patients.

**TABLE S5**. P-values of the seven resource categories subdivided by ten aetiologic groups.

5A. GENERAL WARD NURSING

|  | S. pneumoniae | C. burnetii | H. influenzae | L. pneumophila | Chlamydophila spp. | M. pneumoniae | *Staph. aureus* | Viral | Other |
| --- | --- | --- | --- | --- | --- | --- | --- | --- | --- |
| S. pneumoniae |  |  |  |  |  |  |  |  |  |
| Coxiella burnetii | **<0.001** |  |  |  |  |  |  |  |  |
| H. influenzae | 0.44 | **0.04** |  |  |  |  |  |  |  |
| L. pneumophila | 0.93 | **0.04** | 0.46 |  |  |  |  |  |  |
| Chlamydophila spp. | 0.74 | 0.06 | 0.23 | 0.85 |  |  |  |  |  |
| M. pneumoniae | **0.01** | 0.61 | 0.06 | 0.13 | 0.06 |  |  |  |  |
| *Staph. aureus* | 0.33 | **0.01** | **0.04** | 0.78 | 0.51 | **0.02** |  |  |  |
| Viral | 0.44 | **0.01** | 0.81 | 0.59 | 0.36 | **0.02** | 0.07 |  |  |
| Other | 0.68 | **0.05** | 0.81 | 0.97 | 0.68 | 0.13 | 0.42 | 0.92 |  |
| No pathogen | 0.16 | **0.003** | 0.91 | 0.51 | 0.31 | **0.05** | 0.23 | 0.88 | 0.70 |

Bald numbers indicate a significant difference (p-value <0.05).

Abbreviations: *S. pneumoniae, Streptococcus pneumoniae*; *H. influenzae, Haemophilus influenzae; L. pneumophila, Legionella pneumophila; M. pneumoniaa, Mycoplasma pneumonia;* spp, species; *Staph. aureus, Staphylococcus aureus.*

5B. CLINICAL CHEMISTRY LABORATORY TESTS

|  | S. pneumoniae | C. burnetii | H. influenzae | L. pneumophila | Chlamydophila spp. | M. pneumoniae | *Staph. aureus* | Viral | Other |
| --- | --- | --- | --- | --- | --- | --- | --- | --- | --- |
| S. pneumoniae |  |  |  |  |  |  |  |  |  |
| Coxiella burnetii | **<0.001** |  |  |  |  |  |  |  |  |
| H. influenzae | 0.94 | **0.003** |  |  |  |  |  |  |  |
| L. pneumophila | 0.73 | **<0.001** | 0.64 |  |  |  |  |  |  |
| Chlamydophila spp. | 0.18 | **<0.001** | 0.38 | 0.52 |  |  |  |  |  |
| M. pneumoniae | 0.37 | 0.21 | 0.46 | 0.36 | 0.14 |  |  |  |  |
| *Staph. aureus* | 0.58 | **0.02** | 0.72 | 0.85 | 0.93 | 0.46 |  |  |  |
| Viral | 0.60 | **<0.001** | 0.95 | 0.89 | 0.32 | 0.24 | 0.79 |  |  |
| Other | 0.99 | **0.01** | 0.86 | 0.89 | 0.43 | 0.46 | 0.53 | 0.82 |  |
| No pathogen | 0.45 | **<0.001** | 0.68 | 0.40 | 0.07 | 0.51 | 0.48 | 0.27 | 0.79 |

Bald numbers indicate a significant difference (p-value <0.05).

Abbreviations: *S. pneumoniae, Streptococcus pneumoniae*; *H. influenzae, Haemophilus influenzae; L. pneumophila, Legionella pneumophila; M. pneumoniae, Mycoplasma pneumonia;* spp, species; *Staph. aureus, Staphylococcus aureus.*

5C. MEDICATION DRUGS

|  | S. pneumoniae | C. burnetii | H. influenzae | L. pneumophila | Chlamydophila spp. | M. pneumoniae | *Staph. aureus* | Viral | Other |
| --- | --- | --- | --- | --- | --- | --- | --- | --- | --- |
| S. pneumoniae |  |  |  |  |  |  |  |  |  |
| Coxiella burnetii | **0.04** |  |  |  |  |  |  |  |  |
| H. influenzae | **0.01** | 0.79 |  |  |  |  |  |  |  |
| L. pneumophila | **0.001** | **<0.001** | **0.001** |  |  |  |  |  |  |
| Chlamydophila spp. | 0.26 | **0.04** | **0.01** | 0.08 |  |  |  |  |  |
| M. pneumoniae | 0.07 | 0.62 | 0.60 | **0.01** | **0.05** |  |  |  |  |
| *Staph. aureus* | 0.68 | 0.17 | 0.40 | 0.71 | 1.00 | 0.14 |  |  |  |
| Viral | 0.87 | 0.10 | **0.03** | **0.003** | 0.28 | 0.07 | 0.68 |  |  |
| Other | 0.89 | 0.13 | 0.13 | **0.03** | 0.41 | 0.09 | 0.79 | 0.82 |  |
| No pathogen | 0.26 | 0.08 | 0.07 | **<0.001** | 0.11 | 0.12 | 0.51 | 0.56 | 0.64 |

Bald numbers indicate a significant difference (p-value <0.05).

Abbreviations: *S. pneumoniae, Streptococcus pneumoniae*; *H. influenzae, Haemophilus influenzae; L. pneumophila, Legionella pneumophila; M. pneumoniae, Mycoplasma pneumonia;* spp, species; *Staph. aureus, Staphylococcus aureus.*

5D. MICROBIOLOGY EXAMS

|  | S. pneumoniae | C. burnetii | H. influenzae | L. pneumophila | Chlamydophila spp. | M. pneumoniae | *Staph. aureus* | Viral | Other |
| --- | --- | --- | --- | --- | --- | --- | --- | --- | --- |
| S. pneumoniae |  |  |  |  |  |  |  |  |  |
| Coxiella burnetii | 0.32 |  |  |  |  |  |  |  |  |
| H. influenzae | 0.70 | 0.22 |  |  |  |  |  |  |  |
| L. pneumophila | 0.57 | 0.88 | 0.23 |  |  |  |  |  |  |
| Chlamydophila spp. | 0.85 | 0.66 | 0.52 | 0.82 |  |  |  |  |  |
| M. pneumoniae | 0.86 | 0.76 | 0.67 | 0.80 | 0.80 |  |  |  |  |
| *Staph. aureus* | 0.34 | 0.21 | 0.54 | 0.22 | 0.35 | 0.62 |  |  |  |
| Viral | **0.02** | 0.13 | **0.02** | 0.06 | 0.07 | 0.20 | 0.08 |  |  |
| Other | 0.06 | **0.02** | 0.11 | **0.03** | 0.12 | 0.29 | 0.79 | **0.001** |  |
| No pathogen | **0.001** | 0.16 | 0.06 | 0.11 | 0.07 | 0.28 | 0.06 | 0.41 | **<0.001** |

Bald numbers indicate a significant difference (p-value <0.05).

Abbreviations: *S. pneumoniae, Streptococcus pneumoniae*; *H. influenzae, Haemophilus influenzae; L. pneumophila, Legionella pneumophila; M. pneumoniae, Mycoplasma pneumonia;* spp, species; *Staph. aureus, Staphylococcus aureus.*

5E. RADIOLOGY EXAMS

|  | S. pneumoniae | C. burnetii | H. influenzae | L. pneumophila | Chlamydophila spp. | M. pneumoniae | *Staph. aureus* | Viral | Other |
| --- | --- | --- | --- | --- | --- | --- | --- | --- | --- |
| S. pneumoniae |  |  |  |  |  |  |  |  |  |
| Coxiella burnetii | **<0.001** |  |  |  |  |  |  |  |  |
| H. influenzae | 0.24 | 0.06 |  |  |  |  |  |  |  |
| L. pneumophila | 0.15 | 0.06 | 0.92 |  |  |  |  |  |  |
| Chlamydophila spp. | 0.44 | **0.01** | 0.73 | 0.58 |  |  |  |  |  |
| M. pneumoniae | 0.3 | 0.21 | 0.82 | 0.93 | 0.54 |  |  |  |  |
| *Staph. aureus* | 0.61 | 0.09 | 0.38 | 0.33 | 0.49 | 0.44 |  |  |  |
| Viral | **0.05** | **0.02** | 0.87 | 0.89 | 0.51 | 0.89 | 0.36 |  |  |
| Other | 0.86 | **0.02** | 0.52 | 0.47 | 0.75 | 0.57 | 0.62 | 0.39 |  |
| No pathogen | **0.04** | **0.002** | 0.86 | 0.73 | 0.75 | 0.74 | 0.38 | 0.61 | 0.44 |

Bald numbers indicate a significant difference (p-value <0.05).

Abbreviations: *S. pneumoniae, Streptococcus pneumoniae*; *H. influenzae, Haemophilus influenzae; L. pneumophila, Legionella pneumophila; M. pneumoniae, Mycoplasma pneumonia;* spp, species; *Staph. aureus, Staphylococcus aureus.*

5F. OTHER

|  | S. pneumoniae | C. burnetii | H. influenzae | L. pneumophila | Chlamydophila spp. | M. pneumoniae | *Staph. aureus* | Viral | Other |
| --- | --- | --- | --- | --- | --- | --- | --- | --- | --- |
| S. pneumoniae |  |  |  |  |  |  |  |  |  |
| Coxiella burnetii | **0.01** |  |  |  |  |  |  |  |  |
| H. influenzae | 0.21 | 0.52 |  |  |  |  |  |  |  |
| L. pneumophila | 0.45 | **0.01** | 0.11 |  |  |  |  |  |  |
| Chlamydophila spp. | 0.69 | **0.02** | 0.13 | 0.60 |  |  |  |  |  |
| M. pneumoniae | 0.69 | 0.17 | 0.39 | 0.25 | 0.52 |  |  |  |  |
| *Staph. aureus* | 0.14 | 0.06 | **0.05** | 0.72 | 0.36 | 0.11 |  |  |  |
| Viral | 0.18 | 0.23 | 0.71 | **0.05** | 0.11 | 0.43 | **0.02** |  |  |
| Other | 0.48 | **0.04** | 0.31 | 0.88 | 0.77 | 0.49 | 0.59 | 0.19 |  |
| No pathogen | 0.28 | **0.02** | 0.23 | 0.09 | 0.27 | 0.89 | 0.05 | 0.26 | 0.22 |

Bald numbers indicate a significant difference (p-value <0.05).

Abbreviations: *S. pneumoniae, Streptococcus pneumoniae*; *H. influenzae, Haemophilus influenzae; L. pneumophila, Legionella pneumophila; M. pneumoniae, Mycoplasma pneumonia;* spp, species; *Staph. aureus, Staphylococcus aureus.*

5G. INTENSIVE CARE UNIT NURSING

|  | S. pneumoniae | C. burnetii | H. influenzae | L. pneumophila | Chlamydophila spp. | M. pneumoniae | *Staph. aureus* | Viral | | Other |
| --- | --- | --- | --- | --- | --- | --- | --- | --- | --- | --- |
| S. pneumoniae |  |  |  |  |  |  |  |  | |  |
| Coxiella burnetii | - |  |  |  |  |  |  |  | |  |
| H. influenzae | - | - |  |  |  |  |  |  | |  |
| L. pneumophila | 0.38 | - | - |  |  |  |  |  | |  |
| Chlamydophila spp. | 0.85 | - | - | 1.00 |  |  |  |  | |  |
| M. pneumoniae | - | - | - | - | - |  |  |  | |  |
| *Staph. aureus* | 0.14 | - | - | 0.32 | 0.22 | - |  |  | |  |
| Viral | - | - | - | - | - | - | - | |  |  |
| Other | 0.90 | - | - | 0.72 | 0.81 | - | 0.48 | | - |  |
| No pathogen | 0.50 | - | - | 0.21 | 0.81 | - | 0.11 | | - | 1.00 |

Abbreviations: *S. pneumoniae, Streptococcus pneumoniae*; *H. influenzae, Haemophilus influenzae; L. pneumophila, Legionella pneumophila; M. pneumoniae, Mycoplasma pneumonia;* spp, species; *Staph. aureus, Staphylococcus aureus.*

**TABLE S6.** Median total costs of hospitalisation in euro of pneumococcal vaccines serotypes compared with costs of non-vaccine serotypes, with serotyping determined in two manners.

|  | SPUTUM OR BLOOD SEROTYPES | | | SEROCONVERSION SEROTYPES | | |
| --- | --- | --- | --- | --- | --- | --- |
|  | n | Median costs in euro (IQR) | p-value | N | Median costs in euro (IQR) | p-value |
| 7-valent | 6 | 3,115 (2,908-8,839) |  | 12 | 4,759 (3,058-6,618) |  |
| Other serotypes | 21 | 3,837 (3,221-10,165) | 0.35 | 26 | 4,060 (2,915-5,884) | 0.55 |
| 10-valent | 15 | 3,796 (3,050-14,245) |  | 18 | 3,843 (2,938-5,442) |  |
| Other serotypes | 12 | 3,836 (3,217-5,583) | 0.77 | 20 | 4,189 (3,387-6,177) | 0.50 |
| 13-valent | 17 | 3,835 (3,066-13,869) |  | 26 | 4,198 (2,938-5,442) |  |
| Other serotypes | 10 | 3,684 (3,087-5,040) | 0.58 | 12 | 4,189 (3,387-6,667) | 0.51 |
| 23-valent | 22 | 3,815 (3,074-13,681) |  | 38 | 4,189 (3,027-5,903) |  |
| Other serotypes | 5 | 3,837 (2,987-4,767) | 0.53 | 0 | n/a | n/a |

Abbreviations: IQR, interquartile range; n/a, not applicable; n, number.

Pneumococcal serotypes were determined in two manners: first, positive pneumococcal sputum or blood samples were serotyped by the Quellung reaction. Second, a serotype specific rise in *S. pneumoniae* antibodies against pneumococcal polysaccharides was measured on a Luminex platform (Luminex Corporation, Austin, TX), using a quantitative multiplex immunoassay: the xMAP pneumococcal immunity panel.

7-valent conjugate vaccine contains serotypes 4, 6B, 9V, 14, 18C, 19F, and 23F;

10-valent conjugate vaccine contains serotypes 1, 4, 5, 6B, 7F 9V, 14, 18C, 19F, and 23F;

13-valent conjugate vaccine contains serotypes 1, 3, 4, 5, 6A, 6B, 7F, 9V, 14, 18C, 19A, 19F, and 23F;

23-valent polysaccharide vaccine contains serotypes 1, 2, 3, 4, 5, 6B, 7F, 8, 9N, 9V, 10A, 11A, 12F, 14, 15B, 17F, 18C, 19F, 19A, 20, 22F, 23F, and 3.
